# MiR-125a Is a critical modulator for neutrophil development

**DOI:** 10.1371/journal.pgen.1007027

**Published:** 2017-10-04

**Authors:** Yuting Qin, Lingling Wu, Ye Ouyang, Ping Zhou, Haibo Zhou, Yan Wang, Jianyang Ma, Jinsong Zhang, Yanan Chen, Jie Qian, Yuanjia Tang, Nan Shen

**Affiliations:** 1 Department of Rheumatology and Shanghai Institute of Rheumatology, Renji Hospital, Shanghai Jiao Tong University School of Medicine, Shanghai, China; 2 Institute of Health Sciences, Shanghai Institutes for Biological Sciences, Chinese Academy of Sciences and Shanghai Jiao Tong University School of Medicine, Shanghai, China; 3 State Key Laboratory of Oncogenes and Related Genes, Shanghai Cancer Institute, Renji Hospital, Shanghai, China; 4 Collaborative Innovation Center for Translational Medicine, Shanghai Jiao Tong University School of Medicine, Shanghai, China; 5 The Center for Autoimmune Genomics and Etiology (CAGE), Cincinnati Children's Hospital Medical Center, Cincinnati, OH, United States of America; 6 China–Australia Centre for Personalised Immunology, Renji Hospital, Shanghai Jiao Tong University School of Medicine, Shanghai, China; Centre for Cancer Biology, SA Pathology, AUSTRALIA

## Abstract

MicroRNAs are universal post-transcriptional regulators in genomes. They have the ability of buffering gene expressional programs, contributing to robustness of biological systems and playing important roles in development, physiology and diseases. Here, we identified a microRNA, miR-125a, as a positive regulator of granulopoiesis. *MiR125a* knockout mice show reduced infiltration of neutrophils in the lung and alleviated tissue destruction after endotoxin challenge as a consequence of decreased neutrophil numbers. Furthermore, we demonstrated that this significant reduction of neutrophils was due to impaired development of granulocyte precursors to mature neutrophils in an intrinsic manner. We showed that *Socs3*, a critical repressor for granulopoiesis, was a target of miR-125a. Overall, our study revealed a new microRNA regulating granulocyte development and supported a model in which miR-125a acted as a fine-tuner of granulopoiesis.

## Author summary

MicroRNAs are critical epigenetic modulators in development, physiology and disease processes. Many miRNAs are involved in immune cell development and function, like miR-150 for B cells, miR-181a for T cells. However, studies of miRNAs involvement in granulocyte development and function and related diseases are still limited. In this study, we developed engineering *MiR125a* knockout mice to study the function of miR-125a *in vivo*. We identified *MiR125a* knockout mice had decreased neutrophil numbers and reduced infiltration of neutrophils in the lung in LPS shock model. We deduced that this significant reduction of neutrophils was due to impaired development of granulocyte precursors to mature neutrophils in an intrinsic manner. Furthermore, we demonstrated that *Socs3*, a major repressor that negatively regulates granulocyte development, was a target of miR-125a. This finding not only reveals a new microRNA involving granulocyte development, but also provides insights into the new mechanism of miR-125a during action in endotoxemia.

## Introduction

Neutrophils, also known as polymorphonuclear leukocytes (PMNs), are the most abundant granulocytes which play a crucial role in immune defense and inflammatory reaction. Given that the post-mitotic nature of mature neutrophils, they have short lives about only a few days [1] and need to be regenerated constantly through granulopoiesis, a part of hematopoiesis occurring in the bone marrow of adult mammals. During granulopoiesis, hematopoietic stem cells, at the top of the hematopoietic hierarchy, produce multilineage progenitors and precursors-common myeloid progenitors (CMP) and subsequently granulocyte-monocyte progenitors (GMP) which differentiate into mature granulocytes including eosinophils, basophils and neutrophils [2]. In general, granulopoiesis is in a basal physiological condition. However, emergency granulopoiesis can be rapidly induced to produce large number of neutrophils if severe systemic infection occurs [3].

Hematopoiesis is regulated by a group of cytokines. G-CSF is one of the major cytokine that regulates cell proliferation, differentiation and survival during the neutrophil lineage commitment [4, 5]. The receptor of G-CSF is mainly expressed in granulocytic progenitor cells and mature neutrophils [6].The binding of G-CSF to its receptor triggers receptor dimerization and tyrosine phosphorylation of JAK1, JAK2 and TYK2, which belong to the Janus family of protein tyrosine kinases (JAKs) [7]. These then phosphorylate residues in the cytosolic part of the G-CSF receptor and subsequently activate mitogen-activated protein (MAP) kinase like ERK pathway [8] and the signal transducers and activators of transcription (STATs) including STAT1 and STAT3 (4, 10). SOCS3, as the major repressor of G-CSF signaling, belongs to the suppressor of cytokine signaling (SOCS) family of proteins [9], which can be recruited to phosphorylated cytokine receptors and inhibit JAK catalytic activity and subsequently inhibit activation of ERK and STATs. Moreover, mice with *Socs3* conditionally knocked out in hematopoietic cells [10, 11] develop neutrophilia and inflammatory pathologies.

MicroRNAs (miRs or miRNAs) are universal post-transcriptional regulators in animals and plants. Primary miRNAs are first transcribed by RNA polymerase II or III and are then excised to mature miRNAs (~22 nucleotide) that bind to 3’ untranslated regions (UTR) of their target mRNAs to silence gene expression [12]. More than 1000 miRNA genes have been identified in mammalian genomes [13]. And over 60% of protein-coding genes could be targeted by miRNAs according to computational prediction [14]. Due to their specific features, miRNAs have the ability of buffering gene expression programs and contributing to the robustness of biological systems [15]. Thus they play important regulatory roles in different biological processes. Decades of researches have shown that miRNAs involve in mammalian blood cell development and function [16]. For instance, miR-181a was found to modulate T cell selection [17] and miR-150 was identified as a controller of B cell development [18–20] as well as megakaryocytic versus erythrocytic lineage commitment [21]. In addition, miR-223, which was found highly expressed in neutrophils, played a role in regulating the proliferation of granulocyte progenitors and also mediated the inflammatory function of neutrophils [22, 23].

MiR-125a and miR-125b belong to the miR-125 family, which play a crucial role in many different cellular processes including cell differentiation, proliferation and apoptosis [24]. In order to systematically study the function of miR-125a *in vivo*, we developed miR-125a knockout mice. We examined the hematopoiesis of these mice and found fewer neutrophils in both bone marrow and peripheral blood in the absence of miR-125a. As a consequence of decreased number of neutrophils, *MiR125a* knockout mice were demonstrated with reduced infiltration of neutrophils in the lung and alleviated tissue destruction in an endotoxin challenge model. Furthermore, we found out that the reduction of neutrophils was due to impaired proliferation of immature granulocyte to mature neutrophils in an intrinsic manner. We showed that *Socs3*, a critical repressor for granulopoiesis, was a target of miR-125a. Together, these results suggest that miR-125a is an important regulator of basal granulopoiesis.

## Results

### Decreased numbers but normal function of neutrophils in *MiR125a*^*-/-*^ mice

**Fig 1:**
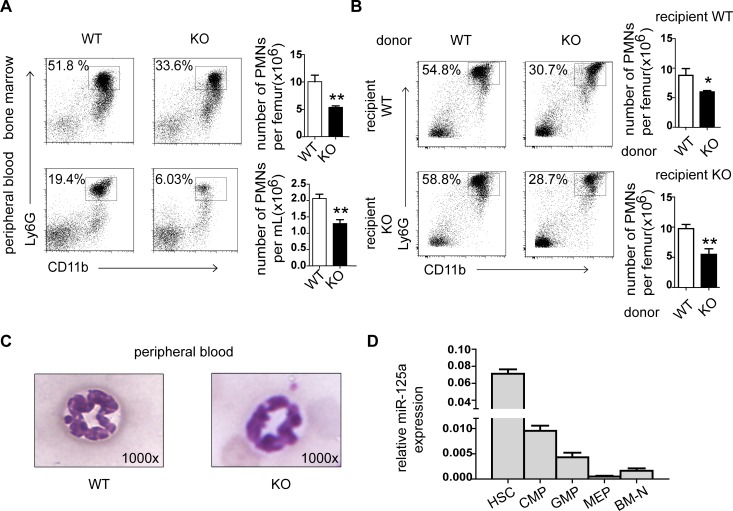
Decreased neutrophils in *MiR125a*^*-/-*^ mice. (A) Flow cytometry analysis of bone marrow (upper panel) and peripheral blood (lower panel). Neutrophils were stained with CD11b-Percp cy5.5 and Ly6G-APC. Bar graphs indicated numbers of neutrophils per femur. Values were represented as mean±s.d., n = 5 mice of each genotype. (B) Flow cytometry analysis of bone marrow neutrophils after bone marrow transplantation for 6 weeks. Bar graphs indicated total numbers of neutrophils. Values were represented as mean±s.d.,n = 5 mice of each genotype. (C) Morphological character of neutrophils in *MiR125a*^*+/+*^and *MiR125a*^*-/-*^ mice. Peripheral blood (original magnification, x1000) of control and knockout mice were stained with Giemsa. (D) Expression of miR-125a during myeloid development (mean±s.d.,n = 3). HSC, hematopoietic stem cells; CMP, common myeloid progenitors; GMP, granulocyte–monocyte progenitors; MEP, megakaryocyte erythroid progenitors; BM-N, bonemarrow neutrophils. **P<0.01, *P<0.05(Student’s *t*-test).

To fully understand the physiological role of miR-125a *in vivo*, we generated the *MiR125a* knockout mice as previously described [25]. These mice are fertile, born at the expected mendelian ratio, and not shown any abnormalities during their growth. However, we found that the white blood cell differential count revealed decreased numbers of neutrophils in *MiR125a*^*-/-*^ mice (1.4 ± 0.3 x 10^6^cells/mL versus 2.2 ± 0.4 x 10^6^ cells/mL) (p<0.0001) while other mature hematopoietic lineage cells including other granulocytes (eosinophils and basophils) were normal (Table 1). Flow cytometry analyses of neutrophils in the bone marrow and peripheral blood confirm these results (Fig 1A). Next we did a bone marrow transfer assay to find out whether reduced granulopoiesis in *MiR125a*^*-/-*^ mice are due to impaired cell-autonomous development or altered cytokine production from the bone marrow stromal cells. We found that decreased number of neutrophils reconstituted with *MiR125a*^*-/-*^ bone marrow cells was both in *MiR125a*^*+/+*^ and *MiR125a*^*-/-*^ recipients (Fig 1B). These results demonstrate that miR-125a contributes to reduced granulopoiesis in a cell-autonomous way. In addition, morphological analysis shows that neutrophils in *MiR125a*^*-/-*^ mice are as mature as those in wild-type mice (Fig 1C). We then examined the expression of miR-125a in different stages of myeloid development and found that miR-125a was highly expressed in hematopoietic stem cells and decreased during maturation of myeloid progenitor cells, indicating that miR-125a may be involved in regulating granulocyte development (Fig 1D).

**Table 1:** Hematological Parameters of *MiR125a*^*-/-*^ mice.

Hematologic cell parameters	*MiR125a*^*+/+*^	*MiR125a*^*-/-*^
WBC(x10^6^/mL)	9.6±3.9	9.2±3.1
RBC(x10^10^/mL)	1.1±0.1	1.1±0.1
Plt (x10^9^/mL)	1.8±0.1	1.7±0.2
White blood cell differential count	*MiR125a*^*+/+*^	*MiR125a*^*-/-*^
Neutrophils(x10^6^/mL)	2.2±0.4	1.4±0.3***
Lymphocytes(x10^6^/mL)	7.1±3.6	7.3±2.8
Monocytes(x10^5^/mL)	4.2±1.3	3.7±1.0
Eosinophils(x10^4^/mL)	2.0±2.2	1.7±1.5
Basophils(x10^4^/mL)	1.5±1.5	2.0±2.9

Peripheral blood from 10-week old *MiR125a*^*+/+*^ and *MiR125a*^*-/-*^ mice was analyzed with HEMAVET 950 animal hematology analyzer. Results represented mean ± s.d., n = 10 of each genotype.

***, P<0.0001 (Student’s *t*-test).

In order to examine whether miR-125a also plays a role in regulating neutrophil function, we tested the ability of activation, migration and killing pathogens between wild-type and *MiR125a*^*-/-*^ neutrophils. Gene expression profiling data of bone marrow neutrophils stimulated with gram-negative bacterial lipopolysaccharide (LPS) showed that most of inflammatory factors and chemokines were induced equally either from *MiR125a*^*-/-*^ or *MiR125a*^*+/+*^ mice (S1A Fig). Then *in vitro* transwell assay showed *MiR125a*^*-/-*^ neutrophils had no detectable abnormality in fMLP or CXCL1 or CXCL2-dependent chemotaxis and migration (S1B Fig). We then used phorbol myristate acetate (PMA) or LPS to stimulate neutrophils and measured the production of reactive oxygen metabolites, which were important for neutrophils to kill pathogens. FACS analysis revealed no difference in the release of reactive oxygen species between wild-type and knock-out neutrophils (S1C Fig). Furthermore *in vitro* killing assay also demonstrated *MiR125a*^*-/-*^ neutrophils had normal ability to clear bacteria and fungi (S1D Fig).

### Lower mortality and neutrophil infiltration in LPS-induced lethal septic shock in *MiR125a*^*-/-*^ mice

**Fig 2:**
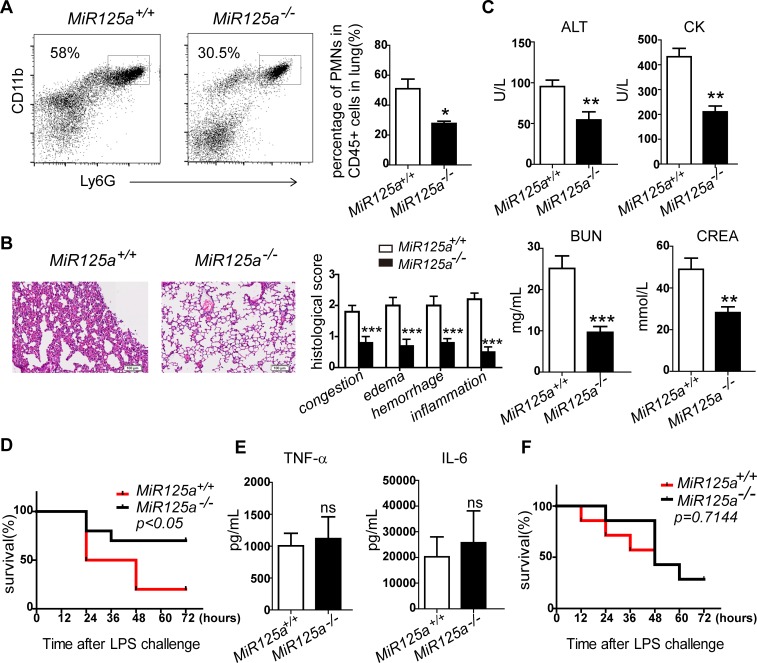
Lower mortality and neutrophil infiltration in LPS-induced lethal septic shock in *MiR125a*^*-/-*^ mice. (A) Flow cytometry analysis of infiltrating neutrophils from lungs of *MiR125a*^*+/+*^and *MiR125a*^*-/-*^ mice challenged with 25 mg/kg LPS after 24 hours. Single cell suspensions of lung cells were previously gated with CD45. Neutrophils were stained with CD11b-Percp cy5.5 and Ly6G-APC. Bar graph shows the average percentage of infiltrating neutrophils (mean±s.d.,n = 3 mice of each genotype). (B) Hematoxylinand-eosin staining of lung sections from WT and KO mice 24 hours after 25 mg/kg LPS injection. Bar graph is the histopathological severity score of lung sections. Histopathological severity of randomly selected fields from the lung sections were graded as 0 (no findings or normal), 1 (mild), 2 (moderate) or 3 (severe) for each of the four parameters(congestion, edema, hemorrhage and inflammation). Theses results were confirmed by a blinded independent researcher. (C) Serum concentrations of aspartate aminotransferase (ALT), blood urea nitrogen (BUN), creatine kinase (CK) and creatinine (CREA) in *MiR125a*^*+/+*^and *MiR125a*^*-/-*^ mice 24 h after injection of 25 mg/kg LPS (mean±s.d.,n = 5 mice of each genotype,). (D) Survival of *MiR125a*^*+/+*^and *MiR125a*^*-/-*^ mice (n = 10 each genotype) intraperitoneally challenged with 45 mg/kg LPS. Data are presented as a Kaplan-Meier plot. P<0.05 (log-rank test). (E) TNF-α and IL-6 concentrations in serum of *MiR125a*^*+/+*^and *MiR125a*^*-/-*^ mice 2h after intraperitoneal injection of 45 mg/kg LPS (mean±s.d., n = 5 mice of each genotype). ns, no significant difference (Student’s *t*-test). (F) Wild-type mice were first depleted of endogenous macrophages by pre-treatment with clodronate liposomes and then were transplanted with 1x10^7^ *MiR125a*^*+/+*^and *MiR125a*^*-/-*^ bone marrow derived macrophages 6 hours before intraperitoneal injection with 45 mg/kg LPS. Survival percentage of these mice are presented as a Kaplan-Meier plot (n = 7 mice of each genotype;p = 0.7114, log-rank test).*P<0.05,**P<0.01, ***P<0.001.

Neutrophils are known to be recruited at inflammatory tissue sites and play a critical role in sepsis and tissue damage [26]. We therefore performed experimental endotoxaemia by injecting a sub-lethal intraperitoneal dose of LPS to *MiR125a*^*-/-*^ mice for 24 hours and measured neutrophil infiltration in the lungs by flow cytometry. Lungs of *MiR125a*^*-/-*^ mice accumulated fewer neutrophils than those of *MiR125a*^*+/+*^ mice (Fig 2A). In addition, we checked the lung sections of *MiR125a*^*-/-*^ and wild-type mice. Consistently with the FACS analysis, lungs of *MiR125a*^*-/-*^ mice show less severe histopathological change, including congestion (hyperplasia of alveolar walls and alveolar collapse), edema (pulmonary interstitial edema), inflammation (neutrophil infiltration) and hemorrhage (engorgement of the capillaries) (Fig 2B). We also found *MiR125a*^*-/-*^ mice had significantly reduced serum amounts of aspartate aminotransferase (ALT), blood urea nitrogen (BUN), creatine kinase (CK) and creatinine (CREA), which were indicators for organ damages (Fig 2C).

We next challenged both *MiR125a*^*-/-*^ and wild-type mice with a lethal dose of LPS. We observed that *MiR125a*^*-/-*^ mice were more resistant to lethal septic shock (Fig 2D). However, serum concentrations of inflammatory cytokine IL-6 and TNF-α during sepsis were similar (Fig 2E). In addition, normal *Il6* and *Tnfa* mRNAs were expressed in peritoneal macrophages and bone marrow-derived macrophages after stimulation with LPS (S2 Fig). To further study whether there is any macrophage involvement, we depleted endogenous macrophages by using clodronate liposomes in wild-type mice and transplanted with *MiR125a*^*+/+*^ or *MiR125a*^*-/-*^ bone marrow-derived macrophages. Then we administrated these mice with the lethal dose of LPS. Results did not show any difference in mortality (Fig 2F). These results implied that cytokine production induced by Toll-like receptors on macrophages did not contribute to resistance to LPS in *MiR125a*^*-/-*^ mice. Thus resistance to a lethal dose of LPS and decreased neutrophils in the lungs with endotoxaemia in *MiR125a*^*-/-*^ mice are likely caused by reduced granulopoiesis.

### Impaired differentiation from granulocyte progenitors to mature neutrophils in *MiR125a*^*-/-*^ mice

**Fig 3:**
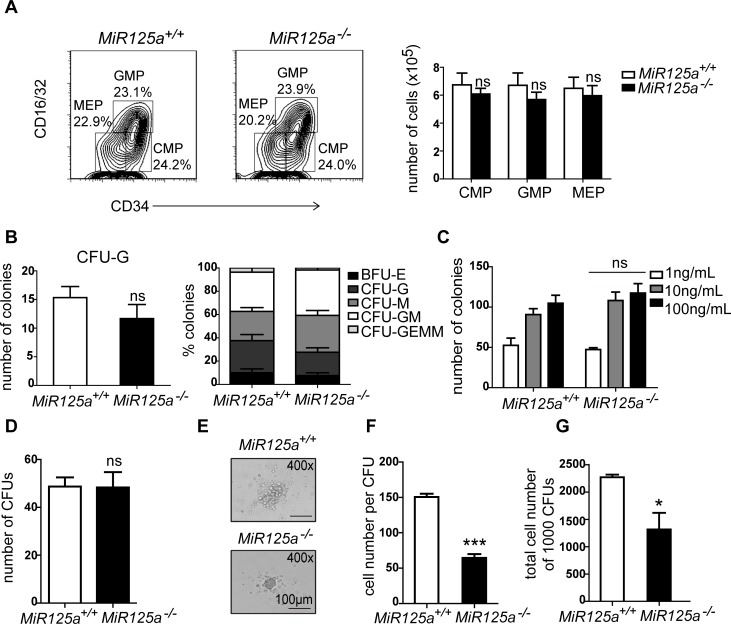
Impaired differentiation from granulocyte progenitors to mature neutrophils in *MiR125a*^*-/-*^ mice. (A) Flow cytometry analysis of myeloid precursor cell populations of 8-week-old mice. Plots shown here were previously gated on Lin^-^Sca-1^-^c-Kit^+^ cells. The right panel shows the overall number of precursors per bone marrow sample isolated from femurs and tibiae (mean±s.d.,n = 6 mice of each genotype). (B-C) Colony numbers of bone marrow cells in methylcellulose colony assays. Myeloid precursors were analyzed in complete methylcellulose medium containing SCF, IL-3, IL-6, and EPO (B) or varying concentrations of G-CSF (C). Values were represented as mean±s.d., n = 3 mice of each genotype. (D-G) 1000 GMPs were sorted from *MiR125a*^*+/+*^ or *MiR125a*^*-/-*^ mice and cultivate in G-CSF containing methylcellulose media. Colony numbers (D), photographed CFUs (E), cell number per CFUs (F) and total cell number of 1000 CFUs (g) were shown. Values were represented as mean±s.d., n = 3 mice of each genotype. ns, none significant difference,*P<0.05, ***P<0.001(Student’s *t*-test).

To study the mechanism of decreased neutrophil numbers in *MiR125a*^*-/-*^ mice, we performed flow cytometry analysis on bone marrow cells in both wild type (WT) and knockout (KO) mice to examine whether the frequency of progenitor cells was disturbed. We found that the numbers of myeloid progenitors did not change (Fig 3A). We then performed colony forming assays on methylcellulose and analyzed them for myeloid precursors in complete medium. There is no significant difference in the frequency of myeloid precursors and numbers of granulocyte colonies (Fig 3B). For greater precision, we performed colony assays in the medium only containing variant concentrations of G-CSF and found that there was also no change in colony numbers (Fig 3C). However, we did notice that colonies from mutant mice were smaller and the cell number in one colony was less than those found in control mice (S3 Fig). Thus we sorted Lin^-^Sca1^-^c-Kit^+^CD34^hi^CD16/32^hi^ GMPs by FACS and estimated their developmental capacity in a CFU assay. We also found the colony number did not change (Fig 3D) but the colony size and the cell number per colony from *MiR125a*-deficient GMPs decreased in the presence of G-CSF (Fig 3E–3G). Thus, it suggested that the development of granulocyte progenitors might be impaired in *MiR125a*^*-/-*^ mice.

### Normal cell death but decreased proliferation of immature neutrophils in *MiR125a*^*-/-*^ mice

**Fig 4:**
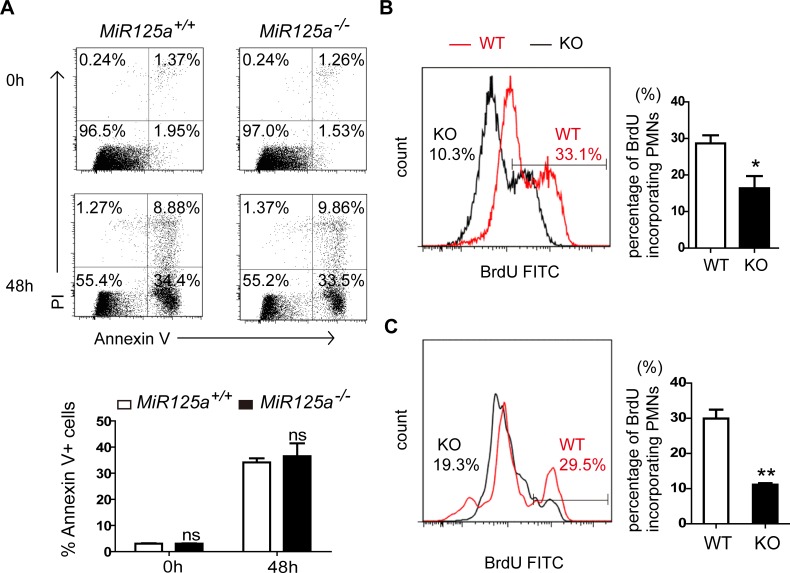
Normal cell death but decreased cell proliferation of in *MiR125a*^-/-^ neutrophils. (A) Apoptosis of bone marrow neutrophils in *MiR125a*^*+/+*^ and *MiR125a*^*-/-*^ mice. 2x10^6^ bone marrow cells were cultured in 10% FBS 1640 medium for 48 hours. Flow cytometry analysis of Ly6G^+^ cell apoptosis by using Annexin V and PI staining. The bar graph shows the percentage of Annexin V + neutrophils (mean±s.d., n = 3). (B-C) Flow cytometry analysis of neutrophils incorporating BrdU in bone marrow (B) and spleen (C) after in vivo pulsing BrdU for 72 hours. Neutrophils were previously gated with CD11b-Percp cy5.5 and Ly6G-APC. Bar graphs indicate the mean percentage of BrdU-incorporating neutrophils (mean±s.d., n = 3). ns, none significant difference, *P<0.05 (Student’s *t*-test).

Since the number of granulocyte progenitors remained unchanged, it would appear that reduction of neutrophils only was due to increased cell death or impaired proliferation from granulocyte progenitors to mature neutrophils. To test the first possibility, we examined cell death rate of Ly6G^hi^ cells from bone marrow by staining them with Annexin V and propidium iodide. We found no difference in the rate of cell death between *MiR125a*^*-/-*^ and wild-type mice (Fig 4A). We then performed *in vivo* BrdU-pulsing assays to analyze neutrophils generation in bone marrow (Fig 4B) and spleen (Fig 4C). Flow cytometry results showed that neutrophils from *MiR125a*^*-/-*^ mice incorporated less BrdU than wild-type mice, indicating that cell proliferation had decreased during the differentiation of granulocyte progenitors into neutrophils.

**Fig 5:**
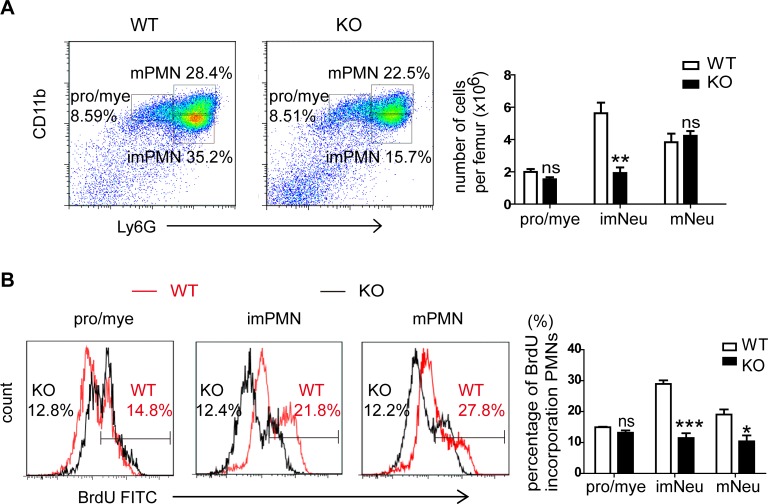
Decreased proliferation of immature neutrophils in *MiR125a*^*-/-*^ mice. (A) Flow cytometry analysis of three subpopulations in CD11b^+^Gr-1^+^ neutrophils in bone marrow. Mature neutrophils (mNeu) indicate CD11b^hi^ Gr-1^hi^ cells. Immature neutrophils (imNeu) indicate CD11b^low^Gr-1^hi^ cells and promyelocytes/myelocytes (pro/mye) indicate CD11b^int^Gr-1^int^ cells. The bar graph shows the average numbers of these subpopulations in *MiR125a*^*+/+*^ and *MiR125a*^*-/-*^ mice. Values were represented as mean±s.d., n = 5 mice of each genotype. (B) Flow cytometry analysis of three populations of CD11b^+^Gr-1^+^ neutrophils incorporating BrdU in bone marrow after in vivo pulsing BrdU for 72 hours. The bar graph indicates the average percentage of intensities of BrdU-incorporating cells (mean±s.d., n = 5). Ns, none significant difference, *P<0.05, ***P<0.001 (Student’s *t*-test).

It has been reported that CD11b^+^ Gr-1^+^ neutrophils in bone marrow are composed of three populations, including CD11b^hi^ Gr-1^hi^ cells (mature Neu), CD11b^low^Gr-1^hi^ cells (immature Neu) and CD11b^int^Gr-1^int^ cells (promyelocytes/myelocytes) [27–29]. According to this, we found the percentage of immature neutrophils was significantly lower in the bone marrow of *MiR125a*-deficient mice while the percentages of promyelocytes/myelocytes and mature neutrophils had no change (Fig 5A). In addition, we found BrdU-incorporating cells in the population of immature and mature neutrophils were significantly lower in *MiR125a* KO mice compared with WT controls while the population of promyelocytes/myelocytes had no change (Fig 5B). Because of post-mitotic nature of mature neutrophils, these BrdU-incorporating mature neutrophils mostly came from BrdU-incorporating immature neutrophils during their last division. Thus we deduced that the neutropenia of *MiR125a*-deficient mice could be due to reduced cell proliferation of CD11b^low^Gr-1^hi^ immature neutrophils.

### Impaired G-CSF signaling in the absence of miR-125a

**Fig 6:**
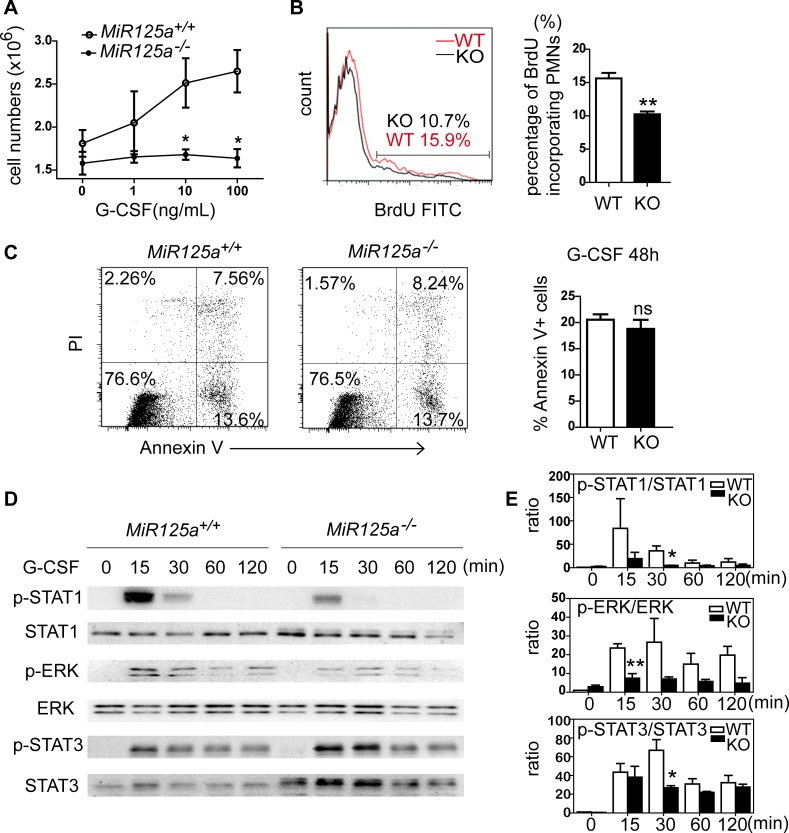
Impaired G-CSF signaling in *MiR125a*^-/-^ neutrophils. (A) Proliferation of bone marrow neutrophils in respond to various concentrations of G-CSF. 2x10^4^ bone marrow neutrophils were cultured with G-CSF in various concentrations for 24 hours. Cell number were then counted. (B) Flow cytometry analysis of BrdU incorporating bone marrow neutrophils in response to G-CSF. Bar graphs show the average percentage of BrdU-incorporating neutrophils. (C) Apoptosis of bone marrow neutrophils in response to G-CSF for 48 hours. The bar graph shows the percentage of Annexin V + neutrophils. (D) Activation of STAT1, ERK and STAT3 in response to G-CSF. Bone marrow neutrophils were stimulated with 10 ng/ml G-CSF for 15 min, 30 min, 60 min and 120 min. Cell lysates were analyzed by immunoblot using antibodies specific for phosphorylated and total STATs, ERK and GAPDH. Representative data are from three independent experiments. (E) The ratio of phosphorylated STAT1, ERK and STAT3 vs. total STAT1, ERK and STAT3. Image J was used to quantitatively analyze the western blots results in (D). All values were represented as mean±s.d., n = 3 mice of each genotype. Ns, none significant difference, ** *P*<0.01, *P <0.05 (Student’s *t*-test).

As G-CSF is the major cytokine during granulocyte differentiation, we purified neutrophils from bone marrow cells and stimulated them with variant concentrations of G-CSF and counted the cell number after 24 hours. We found that the survival number of bone marrow neutrophils from wild-type mice increased substantially with increased G-CSF concentration while bone marrow neutrophils from *MiR125a*^*-/-*^ mice did not increase in number (Fig 6A). We then analyzed apoptosis percentage and BrdU-incorporated cell ratios in response to G-CSF. In accordance with the observation *in vivo*, the amount of BrdU-incorporation was less in the absence of miR-125a (Fig 6B) while the apoptosis percentage has no change (Fig 6C). In addition, we found the mRNA levels of *Gcsfr* and several essential trancriptional factors for granulopoiesis like *Pu*.*1*, *Gata-1*, *Cebpa*, *Cebpb* and *Cebpe* did not change (S4 Fig). These results suggest that decreased cell proliferation in *MiR125a-*deficient mice might be due to impaired G-CSF signaling.

To investigate the molecular mechanism that contributes to impaired G-CSF-dependent proliferation, we examined activation of STAT1, ERK and STAT3 under the G-CSF signaling pathway (Fig 6D). In repeated experiments, we found that the ratio of phosphorylated STAT1, ERK and STAT3 vs. total STAT1, ERK and STAT3 was markedly weaker and less prolonged in different level in *MiR125a*^*-/-*^ neutrophils in response to G-CSF (Fig 6E). This result indicates that the upstream in G-CSF signaling is impaired. However, we noticed that phospho-STAT3 was moderately enhanced while total STAT3 was much higher in *MiR125a*^*-/-*^ bone marrow neutrophils. To determine whether the moderately enhanced p-STAT3 involves in mediating the decreased cell proliferation during maturation of *MiR125a*^*-/-*^ GMPs, we cultured *MiR125a*^*-/-*^ GMPs with G-CSF in the presence of STAT3 inhibitor S3I-201 or DMSO in CFU assays. Results show that inhibiting STAT3 cannot rescue the decelerated cell proliferation of *MiR125a*^*-/-*^ GMP (S5A–S5C Fig). Therefore, according to these data, STAT3 is unlikely to mediate decreased granulocyte differentiation in *MiR125a*^*-/-*^ mice.

### MiR-125a regulates maturation of neutrophils by targeting *Socs3*

**Fig 7:**
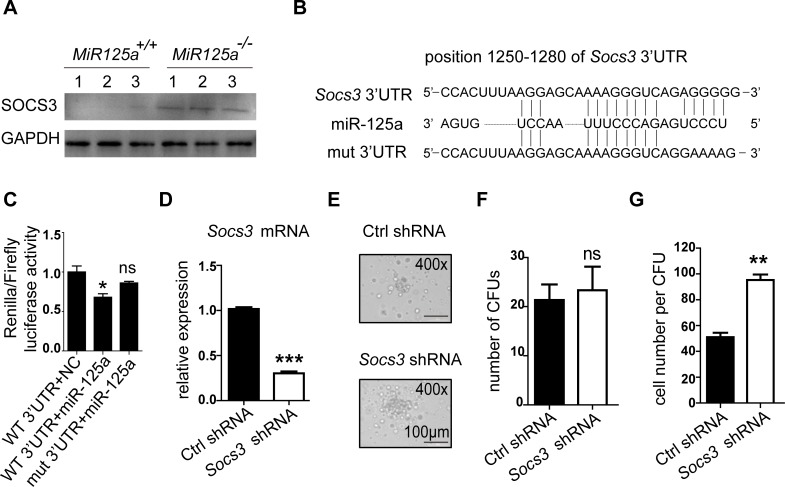
*Socs3* ia a target of miR-125a. (A) Protein expression of SOCS3 in bone marrow neutrophils from *MiR125a*^*+/+*^ and *MiR125a*^*-/-*^ mice. Cell lysates were analyzed by immunoblot using SOCS3 antibody. (B) Schematic presentation of a potential miR-125a binding sites in the 3’UTR regions of Socs3. Sequences below indicate the mutant form of this site. (C) Luciferase reporter gene assay performed on 293T cells transfected with plasmids on which the luciferase reporter gene fused to the fragment of wild-type or mutant 3’UTRs of Socs3. Values were normalized to a firefly gene’s activity on the same construct (mean±s.d., n = 3). (D) The mRNA expression of Socs3 in sorted GFP^+^ GMPs which were transduced with retrovirus of Socs3 shRNA or a control(Ctrl) shRNA. (E-G) 1000 GMPs were sorted from *MiR125a*^*-/-*^ bone marrow lin^-^ cells which were transduced with retrovirus of Socs3 shRNA or a Ctrl shRNA and then cultivated in G-CSF containing methylcellulose media. Photographed CFUs (E), colony numbers (F) and cell number per CFUs (G) were shown. Representative data were from three independent experiments. **P<0.01 (Student’s *t*-test).

**Fig 8:**
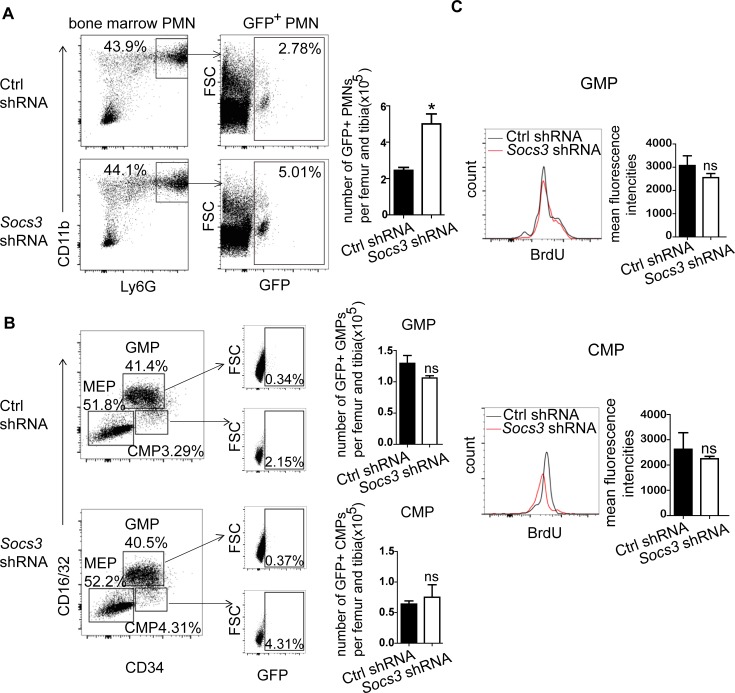
MiR-125a regulates maturation of neutrophils by targeting *Socs3 in vivo*. (A) Flow cytometry analysis of GFP^+^ bone marrow neutrophils after bone marrow transplantation of miR-125a^-/-^ ST-HSCs which are transduced with lentivirus of Socs3 shRNA or a control(Ctrl) shRNA. Bar graphs indicated numbers of GFP^+^ neutrophils per femur and tibia. (B) Flow cytometry analysis of GFP^+^ myeloid precursor cell populations after bone marrow transplantation of miR-125a^-/-^ ST-HSCs which are transduced with lentivirus of Socs3 shRNA or a control(Ctrl) shRNA. Plots shown here were previously gated on Lin^-^Sca-1^-^c-Kit^+^ cells. Bar graphs indicated numbers of GFP^+^ GMPs (upper) or CMPs (lower) per femur and tibia. (C) Flow cytometry analysis of neutrophils incorporating BrdU in bone marrow GFP^+^ GMPs (upper) or CMPs (lower). Bar graphs indicate the mean fluorescence intencities of BrdU-incorporating GMPs (upper) or CMPs (lower). ns, none significant difference, *P<0.05(Student’s *t*-test).

Due to impaired G-CSF signaling pathway in *MiR125a*-deficient mice, we deduced that miR-125a might target a repressor in this signaling. SOCS3 is the major suppressor of G-CSF signaling and neutrophils differentiation [10, 30, 31]. Furthermore, we indeed detected higher SOCS3 protein expression levels in purified neutrophils lacking miR-125a compared to wild-type (Fig 7A). Thus we tested whether miR-125a directly targeted *Socs3*. We firstly predicted possible target sites in 3’UTR of *Socs3* by using RNAhybrid and RNA22, and we found miR-125a has a potential binding site in the 3’UTR of *Socs3* (Fig 7B). Then to confirm whether *Socs3* is targeted by miR-125a, we cloned the full length of the 3’UTR of *Socs*3 onto a construct fused to the renilla reporter gene and mutated the predicted seed sequences. We co-transfected these plasmids with synthetic miR-125a oligonucleotide or negative control oligonucleotide in 293T cells respectively. The results indicated that miR-125a suppressed renilla luciferase activity but the mutants completely inhibited the suppression of the renilla luciferase activity (Fig 7C). These results demonstrate that miR-125a directly targets *Socs3*. But there remains a question whether *Socs3* is a true target of miR-125a to regualte granulopoiesis. To address this issue, we did rescue experiments as follows. Firstly, we used shRNA to knock down *Socs3* expression in *MiR125a*-deficient bone marrow cells and then did CFU assays in the presence of G-CSF. Results are shown that knockdown of *Socs3* decrease *Socs3* mRNA expression (Fig 7D). And the colony size (Fig 7E) and the cell number per colony (Fig 7G) both increase after *Socs3* knockdown. However, the colony number does not change (Fig 7F). Next, we did a *in vivo* rescue assay by isolating short-term hematopoietic stem cells (ST-HSCs) from the bone marrow of *MiR125a* knockout mice, and we transduced these ST-HSCs with concentrated lentivirus of a *Socs3* shRNA or a Ctrl shRNA, both of which contain GFP reporters. Then the transduced cells were collected and injected into the irradiated recipient wild-type mice. Six weeks later, the number of granulocytic progenitors and mature neutrophils was measured by FACS. Consistently with the results of *in vitro* CFU assay, we found that mice transduced with *Socs3* shRNAs had significantly more GFP^+^ bone marrow neutrophils than those transduced with Ctrl shRNAs (Fig 8A). However, the number of GFP^+^ granulocytic progenitor CMPs and GMPs was not affected after *Socs3* inhibition (Fig 8B). Furthermore *in vivo* BrdU-pulsing assays showed that BrdU incorporation of GFP^+^ CMPs and GFP^+^ GMPs did not change after *Socs3* knockdown (Fig 8C). Taken together, both *in vitro* and *in vivo* experiments successfully rescue the decelerated neutrophil development caused by miR-125a deficiency and further confirm that *Socs3* is the main factor of regulating neutrophil development from GMPs to mature neutrophils rather than earlier progenitors in *MiR125a* deficient hematopoiesis.

## Discussion

Previous studies have demonstrated that ectopic expression of miR-125a contributes to expansion of hematopoietic stem cell pool [32, 33]. However, we found an unexpected observation that the numbers of other mature hematopoietic lineage cells were not affected besides neutrophils in *MiR125a* knockout mice (Table 1). These inconsistent results might be explained by the reason that over-expression experiments may lead to gain-of-function phenotypes which cannot be found in knockout mice. Therefore, our results show miR-125a has an indispensable role in regulating neutrophil production.

Neutrophils as well as monocytes-macrophages are the first line of defense in response to systemic inflammation caused by pathogen infection or injury. Under endotoxin challenge, monocytes-macrophages release inflammatory factors such as TNF-α recruiting neutrophils in several organs to mediate tissue destruction [26]. Depletion of neutrophils protects the liver against injury from endotoxin [34]. Thus, like monocyte-macrophages, neutrophils also play a crucial role in endotoxemia. Our study reveals that *MiR125a*^*-/-*^ mice have decreased numbers of neutrophils compared to wild-type mice. In addition, in our LPS shock model, we observed resistance to a lethal dose of LPS in *MiR125a*^*-/-*^ mice but the concentration of TNF-α and IL-6 in the serum remained unchanged compared to control mice. Furthermore, macrophage reconstitution experiments indicated that macrophages did not contribute to resistance to LPS shock in *MiR125a*^*-/-*^ mice. Therefore, we eliminated the possibility that *MiR125a*^*-/-*^ macrophages exhibited less cytokine production in response to stimulation of Toll-like receptors. Importantly, we found less neutrophil infiltration in the lungs and alleviated multiple organ damage in *MiR125a*^*-/-*^ mice after LPS challenge. As we also detected *MiR125a*^*-/-*^ neutrophils were as mature and functional as those in wild-type mice. Therefore, we deduced that resistance to a lethal dose of LPS in *MiR125a*^*-/-*^ mice was mainly due to reduced neutrophil numbers in granulopoiesis.

Granulopoiesis is part of hematopoiesis that maintains the peripheral neutrophil pool steady. In our study, we found *MiR125a* knockout mice showed neutropenia. We considered the main reason for the neutropenia was probably due to decreased cell proliferation from granulocyte progenitors to mature neutrophils in *MiR125a*^*-/-*^ mice. The following are the main evidences demonstrated in this paper. Firstly, numbers of myeloid progenitors including CMPs and GMPs do not change according to FACS and CFU analyses, suggesting miR-125a may not regulate GMPs or even earlier progenitors. Secondly, the colony size is smaller and the cell number per colony is decreased from *MiR125a*-deficient GMPs, implying miR-125a involves in the respectively late stage of granulocyte development. Thirdly, immature and mature neutrophils are incorporated less BrdU in *MiR125a* KO mice while BrdU-incorporating promyelocytes/myelocytes have no change, meaning that miR-125a mediates cell proliferation during the differentiation from immature neutrophils to mature neutrophils. In addition, there is no difference in the rate of cell death between *MiR125a*^*-/-*^ and wild-type mice by staining with Annexin V and propidium iodide, excluding the possibility that miR-125a-mediated cell death of neutrophils. Furthermore, other granulocytes (eosinophils and basophils) are not affected in *MiR125a* knockout mice (Table 1) also indicating that miR-125a is specific for regulating immature neutrophils rather than affecting earlier common granulocyte precursors.

To investigate the molecular mechanism of miR-125a in regulating neutrophil development, we checked the activation of G-CSF signaling pathway in wild-type and *MiR125a* deficient neutrophils. G-CSF is the major growth factor during each developmental stage of granulopoiesis [35]. STAT3, STAT1 and ERK are downstream transcription factors in G-CSF signaling [36]. From western blot analysis, we found *MiR125a* deficiency mainly caused impaired G-CSF signaling pathway through weakening the phosphorylation ratio of downstream transcription factors. But it made us a little bit confused. Although the phosphorylation ratio of STAT3 was reduced, phospho-STAT3 was moderately enhanced while total STAT3 was much higher in *MiR125a*^*-/-*^ neutrophils. In order to solve this problem, we used STAT3 inhibitor S3I-201 in GMP CFU assays. Results demonstrated that inhibiting STAT3 cannot rescue the decelerated differentiation from *MiR125a*^*-/-*^ GMP. Thus we deduce that the phenomenon of the enhanced total STAT3 might be through other unknown mechanisms and it is unlikely to mediate decreased granulocyte differentiation in *MiR125a*^*-/-*^ mice. Owing to the weak G-CSF signaling in *MiR125a*-deficient mice, we deduce that miR-125a might target a repressor in this pathway. SOCS3 is the principal suppressor of G-CSF signaling. It can bind to pY729 of the G-CSF receptor and directly inhibit receptor binding to JAKs, thus repressing downstream signaling [30, 31, 37]. Particularly the mice in which *Socs3* is conditionally knocked-out in bone marrow have increased neutrophil number and enhanced cellular responses to G-CSF including an increase in proliferative capacity [10, 11]. In our study, we actually identified *Socs3* as a direct target of miR-125a. And the expression of *Socs3* was indeed enhanced in *MiR125a*^*-/-*^ neutrophils, weakening G-CSF signaling and eventually reducing neutrophils differentiation (S6 Fig). Furthermore, both *in vivo* and *in vitro* rescue experiments demonstrated that Socs3 indeed was the main target of miR-125a to regulate late stage development of neutrophils rather than earlier progenitors. Nevertheless, we deduce that miR-125a promotes granulopoiesis mainly by targeting suppressor *Socs3*.

MiRNAs are abundant regulators of transcriptional programs. They serve as fine-tuners of biological systems by giving signaling pathways a threshold to protect from unwanted or wrong signals and making signal output more precise and appropriate [38]. In many signaling pathways, the expression of miRNAs can be induced or repressed in response to outside stimuli and form feed-forward or feedback mechanisms with other signaling components [13]. However, basal expression of miRNAs is important for cell-type-specific gene expression through acting as switches like transcriptional factors during cell lineage determination [39]. Hematopoietic lineage differentiation is also switched by miRNAs. For example, miR-150 for B cell [18–20], megakaryocytic and erythrocytic lineage commitment [21], and miR-223 for granulocytic differentiation [22, 23]. In this paper, we proposed a model that miR-125a served as a positive regulator of physiological granulopoiesis by amplifying G-CSF signal strength and duration. In order to get a view of the regulation of miR-125a, we examined whether the expression of miR-125a was also affected by G-CSF signaling. However, we did not detect a significant change of the expression of miR-125a in granulocytes after G-CSF stimuli. As we found that miR-125a was decreased during maturation of granulocytes, we detected the expression of its target *Socs3* which was also down-regulated and the expression of miR-125a and its target *Socs3* exhibited a positive correlation in granulocyte development (S7 Fig). Although this kind of correlation between miRNA and its targets is against the repressive nature of miRNA-mediated gene regulation, bioinformative analysis shows that it is prevalent [40]. Because miRNAs often repress target genes through translational inhibition and have minor effects on target mRNA levels, so miRNAs and their targets levels are mainly controlled by upstream transcription factors [40]. According to this model, both *Socs3* and miR-125a are down-regulated during granulopoiesis and down-regulated miR-125a leads to up-regulated *Socs3* as a feed-forward signal. Thus this circuit can tune upstream signal fluctuation and eventually maintain SOCS3 protein homeostasis. As *Socs3* is a critical negative regulator of granulopoiesis, its level in progenitors of granulocytes can affect the neutrophils differentiation and any significant change may lead to pathological consequences, namely neutrophilia and neutropenia. From this view, miR-125a modulation eventually provides a steady device to maintain differentiation and homeostasis of neutrophils rather than to simply repress the expression of *Socs3*.

In conclusion, we showed that miR-125a can positively regulate granulopoiesis. We demonstrated that miR-125a positively regulated G-CSF-dependent proliferation during the development of granulocytes by targeting *Socs3*. Our findings reveal a new microRNA involving granulocyte development and provide insights into the function of miR-125a during hematopoiesis. Future genetic studies will focus on how miR-125a is regulated during hematopoietic development.

## Materials and methods

### Mice

*MiR125a* knockout mice were generated as previously described [25] and maintained under specific pathogen–free conditions at Institute of Health Sciences, Chinese Academy of Sciences animal breeding facility, according to institute guidelines. 8 to 12-week-old *MiR125a* knockout mice and their littermate controls were used for experiments. All experiments involving mice were in accordance with the Regulations for the Administration of Affairs Concerning Experimental Animals of 1988, issued by the State Scientific and Technological Commission for China. And these experiments were approved by the Biomedical Research Ethics Committee of the Shanghai Institutes for Biological Sciences, Chinese Academy of Sciences.

### Flow cytometry

To analyze neutrophils, single cell suspensions of bone marrow or peripheral blood or spleen were stained with CD11b PerCP-Cyanine5.5 (eBioscience 45-0112-82) and Ly-6G-APC (eBioscience 17-5931-82). To measure neutrophil infiltration in the lung, lung tissues were cut into very small fragments and digested by collagenase and DNase I for 20 minutes at 37°C. Single cell suspensions were then stained with CD45-FITC (BD pharmingen, 553080), Ly-6G-APC and CD11b PerCP-Cy5.5. To detect the myeloid progenitor cells, bone marrow cells were pre-stained with biotin-conjugated mouse lineage panel (BD pharmingen, 559971), and then stained with streptavidin-V450 (BD horizon, 560797), Sca-1-PE-Cy7 (BD pharmingen, 558162), c-Kit-PE (BD pharmingen, 553355), CD34-FITC (BD pharmingen, 560238) and CD16/32-APC (eBioscience, 17-0161-82). Flow cytometry was conducted on a FACS Aria (BD Biosciences).

### Bone marrow transfer assay

The recipient mice were fed with acidic (pH 2.6), antibiotic water for one week before irradiation and then were given 8.0 Gy irradiation by using a ^137^Cesium Gammacell source. 4 hours later, the mice were injected with 2x10^7^ bone marrow cells from the donor mice via tail vein and then were kept on giving acidic antibiotic water for the rest of their lives.

### Cell sorting

To sort hematopoietic stem cells and progenitor cells, bone marrow cells were pre-enriched by depleting lineage positive cells (Stemcell, 19756). Hematopoietic stem cells were then sorted by Sca1^+^c-Kit^+^Lin^-^. CMPs were sorted by Sca1^-^c-kit^+^Lin^-^CD34^+^CD16/32^-^. GMPs were sorted by Sca1^-^c-kit^+^Lin^-^CD34^+^CD16/32^+^ and MEPs were sorted by Sca1^-^c-kit^+^Lin^-^CD34^-^CD16/32^-^. The purity of each cell population reached 95%. Neutrophils were isolated from bone marrow or peritoneal cavity by using the Neutrophil Isolation Kit (Miltenyi Biotec, 130-097-658). The purity of the isolated neutrophils was about 90%, as determined by flow cytometry.

### Microarray analysis

Total RNA was isolated using TRIzol reagent (Life technologies). RNA quality was assessed with an Agilent 2100 Bioanalyzer (Agilent), and only samples with an RNA integrity number > 8 were used. Global mRNA expression in bone marrow neutrophils with or without LPS stimulation samples from and *MiR125a*^+/+^ and *MiR125a*^-/-^ mice were assayed with the Affymetrix GeneChip Mouse Genome 430 2.0 Array. Data were deposited in GEO (GSE63739, http://www.ncbi.nlm.nih.gov/geo/query/acc.cgi?acc=GSE63739) and analyzed with R and the associated BioConductor packages.

### Chemotaxis assays

Isolated bone marrow neutrophils were resuspended in 0.1% BSA 1X Hanks balanced salt solution containing calcium and magnesium (Gibco) and plated in 3 μm Transwells (1X10^5^ cells per Transwell, Corning) in the absence or presence of the indicated chemokine in the lower chamber (0.1 mM fMLP, Sigma; 250 ng/mL MIP-2, Peprotech; 1μg/mL KC, Peprotech). After incubation at 37°C for 3 hours, numbers of cell that migrated through transwell were counted.

### Oxidative burst assays

Isolated bone marrow neutrophils were incubated in the presence of 1 μM dihydrorhodamine (Sigma) during stimulation with different concentrations of PMA (Sigma) for 15 minutes or LPS for 4 hours (Sigma). The oxidative burst of neutrophils was then analyzed by flow cytometry.

### In vitro killing assays

2x10^5^ *Candida albicans* strain SC5314 or 1x10^7^ *Citrobacter rodentium* were incubated with or without 5x10^5^ bone marrow neutrophils in flat-bottom 96-well plates for 4 hours. Then all wells were treated with 0.02% triton-X 100 in PBS for 5 minutes. Surviving bacteria or fungi were incubated with 10μl MTT (5mg/mL) for 4 hours at 37°C then formazans were dissolved in DMSO and fluorescence was measured at 570 nm absorption wavelength.

### Quantitative real-time RT-PCR

Total RNA was isolated with TRIzol reagent (Life Technology). Expression of microRNAs in sorted cell populations was determined by quantitative PCR using the TaqMan MicroRNA Assay (Applied Biosystems). MicroRNA expression was normalized to snoRNA202. *Socs3*, *Il6*, *Tnfa*, *Gcsfr*, *Pu*.*1*, *Gata-1*, *Cebpa*, *Cebpb* and *Cebpe* mRNA expression levels were quantified by using SYBR PrimeScript reverse-transcription–PCR kit (Takara). Expression levels were normalized to endogenous expression of *Gapdh*.

### Macrophage depletion and reconstitution experiments

Wild-type mice were first depleted of endogenous macrophages by pre-treatment with 100 μl clodronate liposome Clophosome-A (FormuMax Scientific) on Day1 and Day2. On Day3, these mice were transplanted with 1x10^7^ *MiR125a* ^+/+^ or *MiR125a* ^-/-^ bone marrow-derived macrophages. Macrophage depletion was detected by flow cytometry on Day3 and Day6 and the spleen and bone marrow macrophages were depleted >90%.

### Colony-forming cell assays

To count the number of GMPs, 5x10^4^ bone marrow cells were cultured in methylcellulose (Mouse Methylcellulose Base Medium, R&D Technologies) added to various concentrations of recombinant murine G-CSF (R&D Technologies). After 10 days, colony numbers were counted. To quantify multi-potential progenitors and lineage-restricted progenitors, 2x10^4^ bone marrow cells were plated in complete methylcellulose medium (Stemcell, 03434). After 12 days, colonies were counted and analyzed morphologically.

### Apoptosis assays

Bone marrow cells were cultured in 10% FBS RPMI 1640 medium (Life Technologies) for 48 hours, washed and stained for Ly-6G-APC and Annexin V FITC and PI (BD Biosciences) and analyzed by flow cytometry.

### BrdU cell incorporation assays

For the *in vivo* BrdU-incorporation experiment, mice aged 8–10 weeks were intraperitoneally injected with 200 μl of a 10mg/mL BrdU solution. After 3 days, mice were sacrificed and the spleen and bone marrow cells were harvested to detect BrdU-positive neutrophils. For *in vitro* BrdU-labeling of cells, bone marrow neutrophils were isolated and stimulated with 100 ng/ml G-CSF for 24 hours followed by incubating cells with 10 μM BrdU for 1 hour. BrdU-positive neutrophils were detected by using the BrdU flow kit from Pharmingen (BD Biosciences, 559619) with a FITC-labeled anti-BrdU antibody. Neutrophils were stained with CD11b-Percp Cy5.5 and Ly-6G-APC before fixation and permeabilization of the cells.

### Protein analysis

TNF-α and IL-6 in mice serum were detected by R&D Technologies duo set ELISA kit. For immunoblotting experiments, bone marrow cells or neutrophils were lysed with RIPA buffer and blotted with indicated antibodies. P-STAT3, STAT3, p-STAT1, STAT1, p-ERK, ERK and SOCS3 were all purchased from Cell Signaling Technology. GAPDH antibodies were obtained from Abcam.

### Luciferase assays

To test whether miR-125a directly target the *Socs3* 3′ UTR, 293T cells were plated in 96-well plates and transfected with 10 ng wild-type or mutant *Socs3* 3′ UTR and the synthetic miR-125a oligonucleotide or negative control oligonucleotide by using Lipofectamine 2000 reagent (Invitrogen). Firefly and renilla luciferase activities were determined after 24 hours using the Dual-Luciferase Reporter Assay System (Promega). The values were normalized to firefly luciferase.

### Virus package and transduction assays

To generate a retrovirus construct, MSCV-LTR miR30-PIG (LMP) plasmids were cloned into *Socs3*-specific hairpin RNA. The target sequence is as follows: CGC GAG TAC CAG CTG GTG GTG A. Plate-E cells were transfected with 30ug LMP shRNA for a dish and retroviruses were harvested from culture supernatant after 48 hours. Mice bone marrow cells were depleted lineage positive cells by magnetic beads, stimulated with G-CSF overnight, then infected with recombinant retrovirus. 48 hours later, green fluorescent protein expressing GMPs were sorted for CFU assays.

To generate a lentivirus construct, pLVX-shRNA2 plasmids were cloned into *Socs3*-specific hairpin RNA. The target sequence is the same as above. 293T cells were transfected with 15ug pLVX-shRNA2 together with 8ug pMD2.G and 15ug psPAX2 plasmids for one dish. Lentivirus were harvested and concentrated from culture supernatant after 72 hours. Bone marrow cells of *MiR125a* ko mice were depleted lineage positive cells by magnetic beads, and short-term hematopoietic stem cells (ST-HSC) were sorted by sca-1^+^c-kit^+^CD135^-^CD34^+^ and resuspended at 1 x 10^4^ in 75 uL StemSpan (StemCell Technologies), supplemented with 50 ng/ml SCF (Peprotech) in a round-bottomed well of a 96-well plate. 2.5 x 10^7^ units of lentivirus were added into each well after 2 hours, predetermined to give about 20% transduction efficiency by measuring of GFP positive cells in pilot experiments. Then plates were spun at 900g for 90 min, and cultured at 37°C with 5% CO2-in-air. Cells were collected and washed after 4.5 hours and per 1.5 x 10^4 ST-HSCs were resuspended in 250 ul PBS which was then injected into each irradiated recipient wild-type mouse.

## Supporting information

S1 Fig Neutrophil function assays in *MiR125a*^*+/+*^ *and MiR125a*^*-/-*^ mice. (A) Heatmap of inflammatory and chemokine genesof neutrophils under sitimulation of LPS. Bone marrow neutrophils from miR-125a deficient and WT mice were stimulated with LPS and harvested in Trizol. Samples from three independent experiments were pooled for the microarray analysis. (B) Number of migrated bone marrow neutrophils in fMLP or CXCL1 or CXCL2-dependent chemotaxis assays (mean ± s.d., n = 3 each genotype). (C) Reactive oxygen species produced by bone marrow neutrophils were measured by FACS analysis of oxidation of dihydrorhodamine 123. Upper panel showed bone marrow neutrophils were stimulated LPS 200ng/mL for 4 hours (red curves) or PBS (black curves). Lower panel showed bone marrow neutrophils were stimulated PMA 50 ng/mL for 15 minutes (red curves) or DMSO (black curves). Bar graphs represented the mean fluorescent intensity of all cells in response to different concentration of LPS or PMA (mean±s.d.,n = 3). (D) *In vitro* killing assay of bone marrow neutrophils from *MiR125a*^+/+^ and *MiR125a*^*-/-*^ mice incubated with Citrobacter rodentium or C. albicans (mean ± s.d.,n = 3 each genotype). Ns, none specific significance (Student’s *t*-test).
(TIF)

S2 Fig Cytokine production of macrophages from *MiR125a*^*-/-*^ mice in response to LPS. Bone marrow-derived macrophages with stimuli of LPS, expression of inflammatory cytokine *Tnfa* (left) and *Il6* (right) mRNA was detected by real-time quantitative PCR (mean±s.d.,n = 3). Ns, none specific significance (Student’s *t*-test).
(TIF)

S3 Fig Morphological character of colonies on methylcellulose. Bone marrow cells from *MiR125a*^*+/+*^ and *MiR125a*^*-/-*^ mice were analyzed for GMPs in methylcellulose medium containing 100ng/ml G-CSF. Colonies were pictured on day 10 (original magnification, 40 X for upper panel; 200 X for lower panel).
(TIF)

S4 Fig The mRNA expression of GCSFR and transcriptional factors in wild-type and *MiR125a* knockout mice. Bone marrow neutrophils were extracted RNA and determined the expression of *Gcsfr*, *Pu*.*1*, *Gata-1*, *Cebpa*, *Cebpb* and *Cebpe* by Real-tme PCR. Ns, none specific significance (Student’s *t*-test).
(TIF)

S5 Fig The role of STAT3 in the neutropenia of *MiR125a-*deficient mice. 1000 GMPs were sorted from *MiR125a*^*-/-*^ bone marrow cells and then cultivated in G-CSF and S3I-201 or DMSO containing methylcellulose media. Photographed CFUs (A), colony numbers (B) and cell number per CFUs (C) were shown. Representative data were from three independent experiments. Ns, none specific significance (Student’s *t*-test).
(TIF)

S6 Fig Overview of G-CSF signaling pathway in *MiR125a*^*+/+*^ and *MiR125a*^*-/-*^ granulocytes. In wild-type granulocytes, miR-125a down-regulates the expression of SOCS3 that was leading to activation of STAT1, STAT3 and ERK. While in *MiR125a* -deficient granulocytes, the expression of SOCS3 was enhanced, weakening of STAT1, STAT3 and ERK activation and eventually reduced granulopoiesis.
(TIF)

S7 Fig The expression and regulation sketch of miR-125a and *Socs3* during granulocytes development. The expression of miR-125a and *Socs3* mRNA was detected by real-time quantitative PCR (mean±s.d.,n = 3) (left). The regulation circuit of miR-125a and *Socs3* during granulocyte development (right).
(TIF)
