# Supplementary figures and images for: MiR-125a Is a critical modulator for neutrophil development
Source: PLoS Genet. 2017 Oct 4;13(10):e1007027. doi: 10.1371/journal.pgen.1007027 (PMC5643141; doi:10.1371/journal.pgen.1007027)

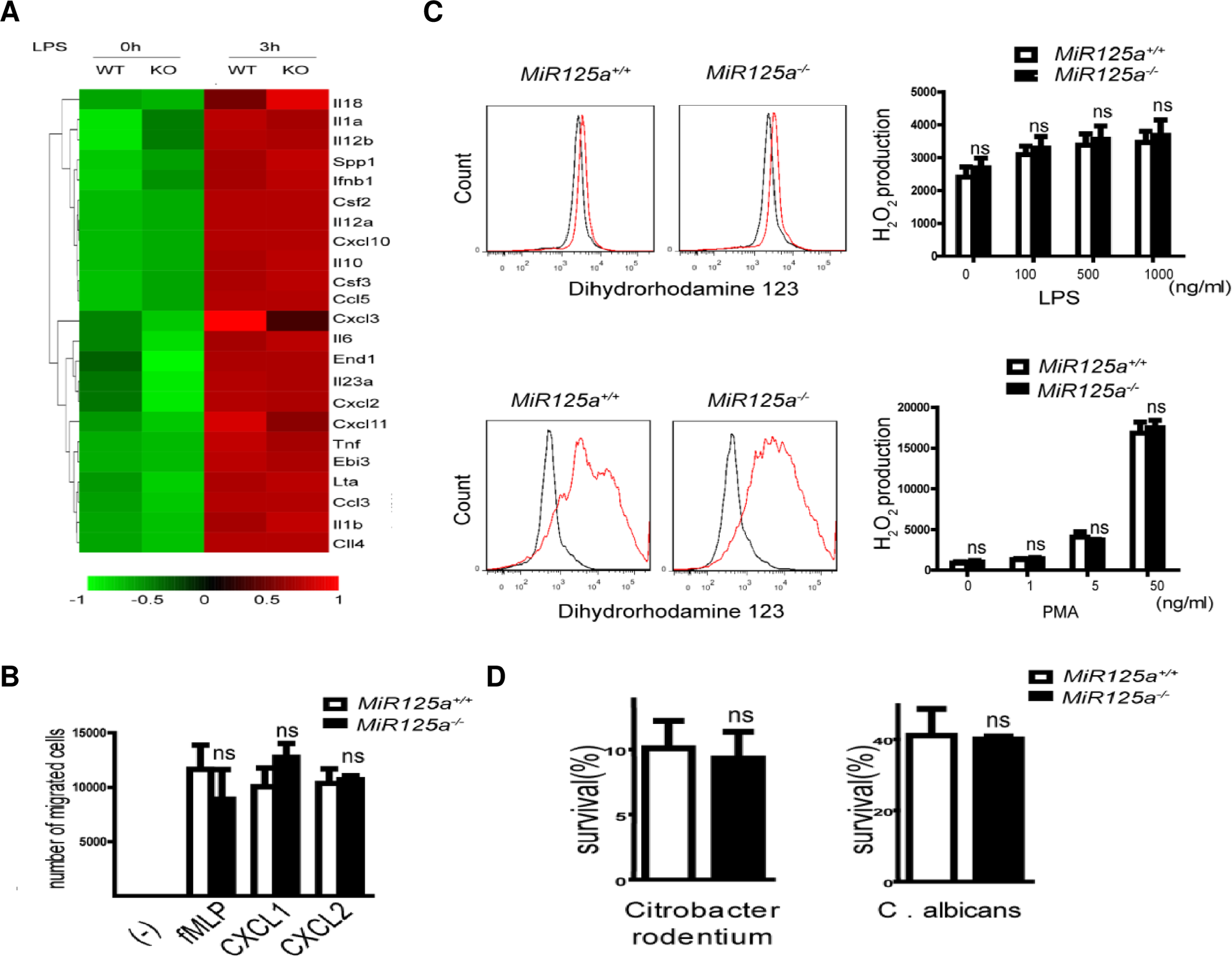

Supplement: S1 Fig — (A) Heatmap of inflammatory and chemokine genesof neutrophils under sitimulation of LPS. Bone marrow neutrophils from miR-125a deficient and WT mice were stimulated with LPS and harvested in Trizol. Samples from three independent experiments were pooled for the microarray analysis. (B) Number of migrated bone marrow neutrophils in fMLP or CXCL1 or CXCL2-dependent chemotaxis assays (mean ± s.d., n = 3 each genotype). (C) Reactive oxygen species produced by bone marrow neutrophils were measured by FACS analysis of oxidation of dihydrorhodamine 123. Upper panel showed bone marrow neutrophils were stimulated LPS 200ng/mL for 4 hours (red curves) or PBS (black curves). Lower panel showed bone marrow neutrophils were stimulated PMA 50 ng/mL for 15 minutes (red curves) or DMSO (black curves). Bar graphs represented the mean fluorescent intensity of all cells in response to different concentration of LPS or PMA (mean±s.d.,n = 3). (D) In vitro killing assay of bone marrow neutrophils from MiR125a+/+ and MiR125a-/- mice incubated with Citrobacter rodentium or C. albicans (mean ± s.d.,n = 3 each genotype). Ns, none specific significance (Student’s t-test). (TIF) [file pgen.1007027.s001.tif]

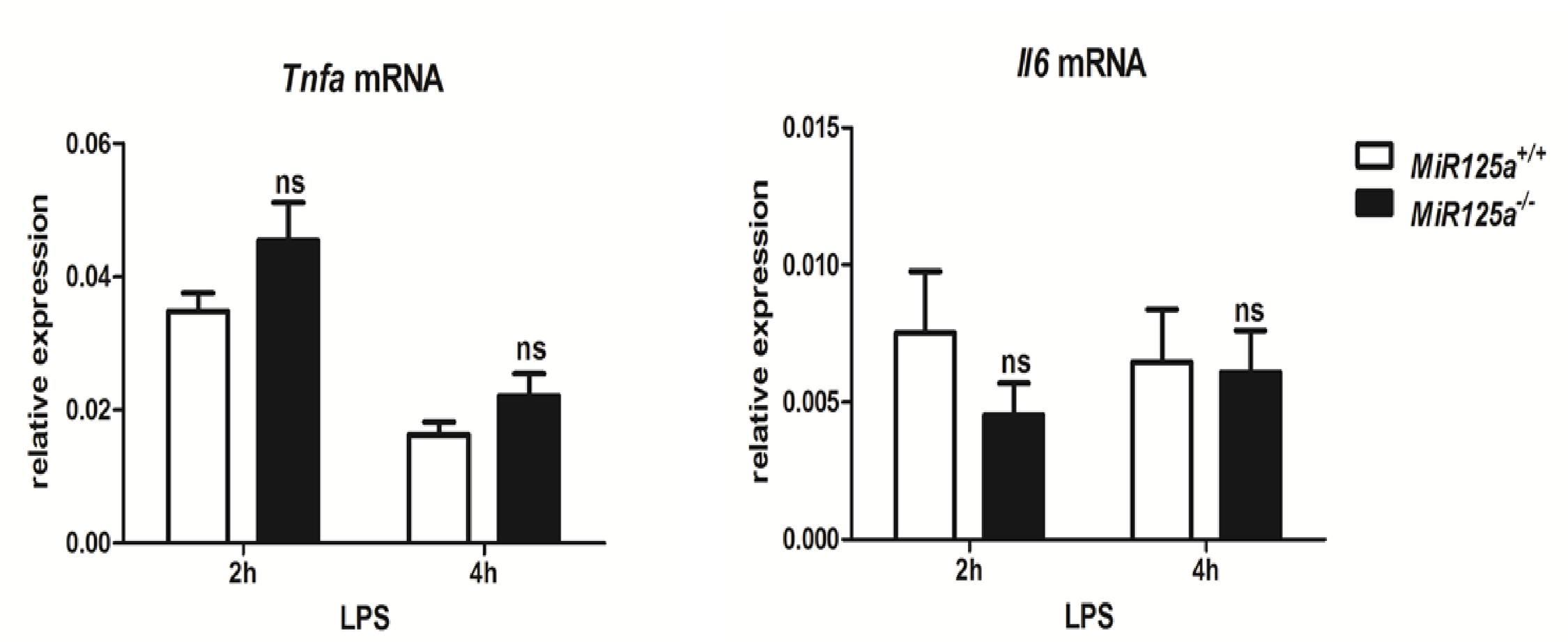

Supplement: S2 Fig — Bone marrow-derived macrophages with stimuli of LPS, expression of inflammatory cytokine Tnfa (left) and Il6 (right) mRNA was detected by real-time quantitative PCR (mean±s.d.,n = 3). Ns, none specific significance (Student’s t-test). (TIF) [file pgen.1007027.s002.tif]

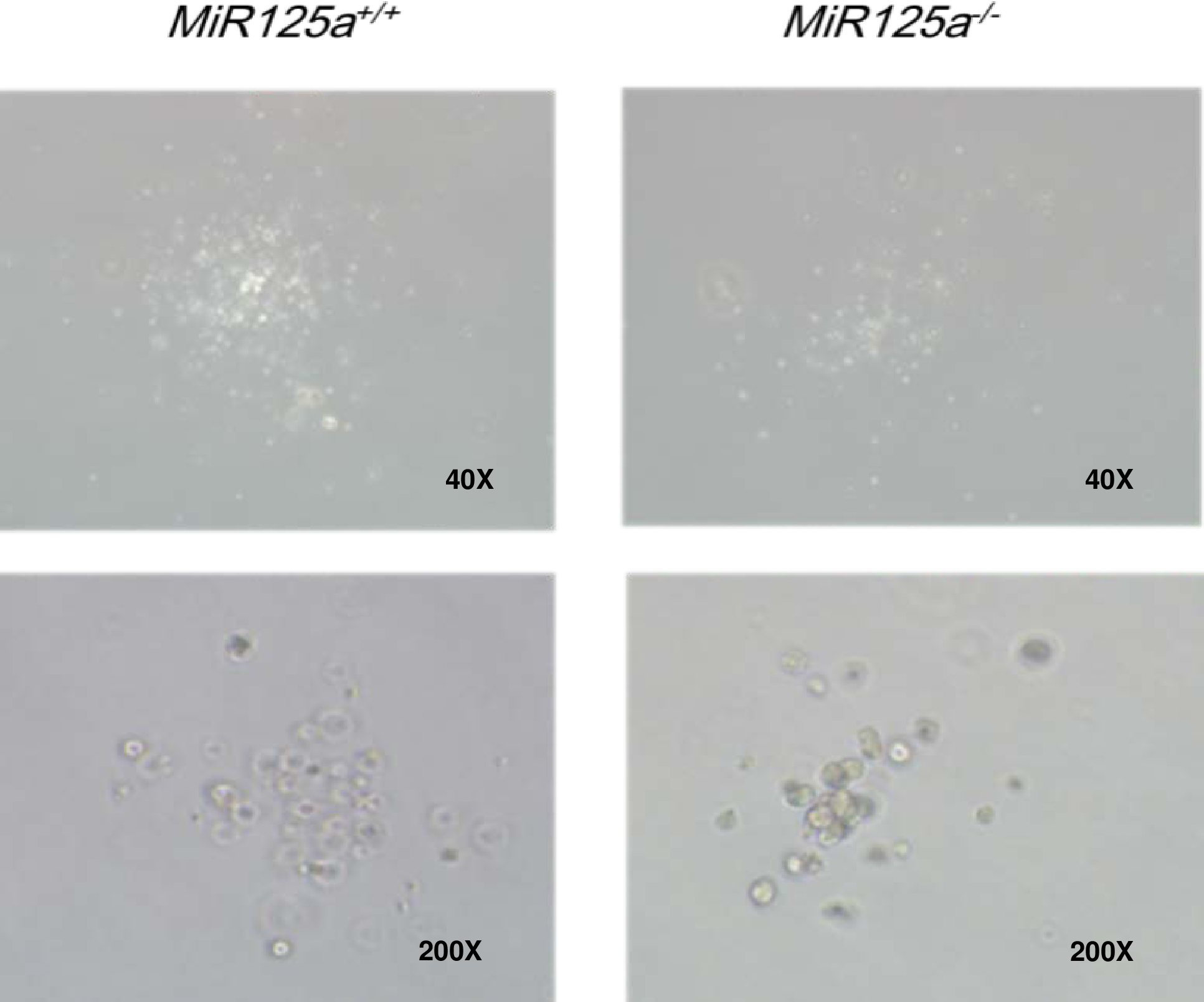

Supplement: S3 Fig — Bone marrow cells from MiR125a+/+ and MiR125a-/- mice were analyzed for GMPs in methylcellulose medium containing 100ng/ml G-CSF. Colonies were pictured on day 10 (original magnification, 40 X for upper panel; 200 X for lower panel). (TIF) [file pgen.1007027.s003.tif]

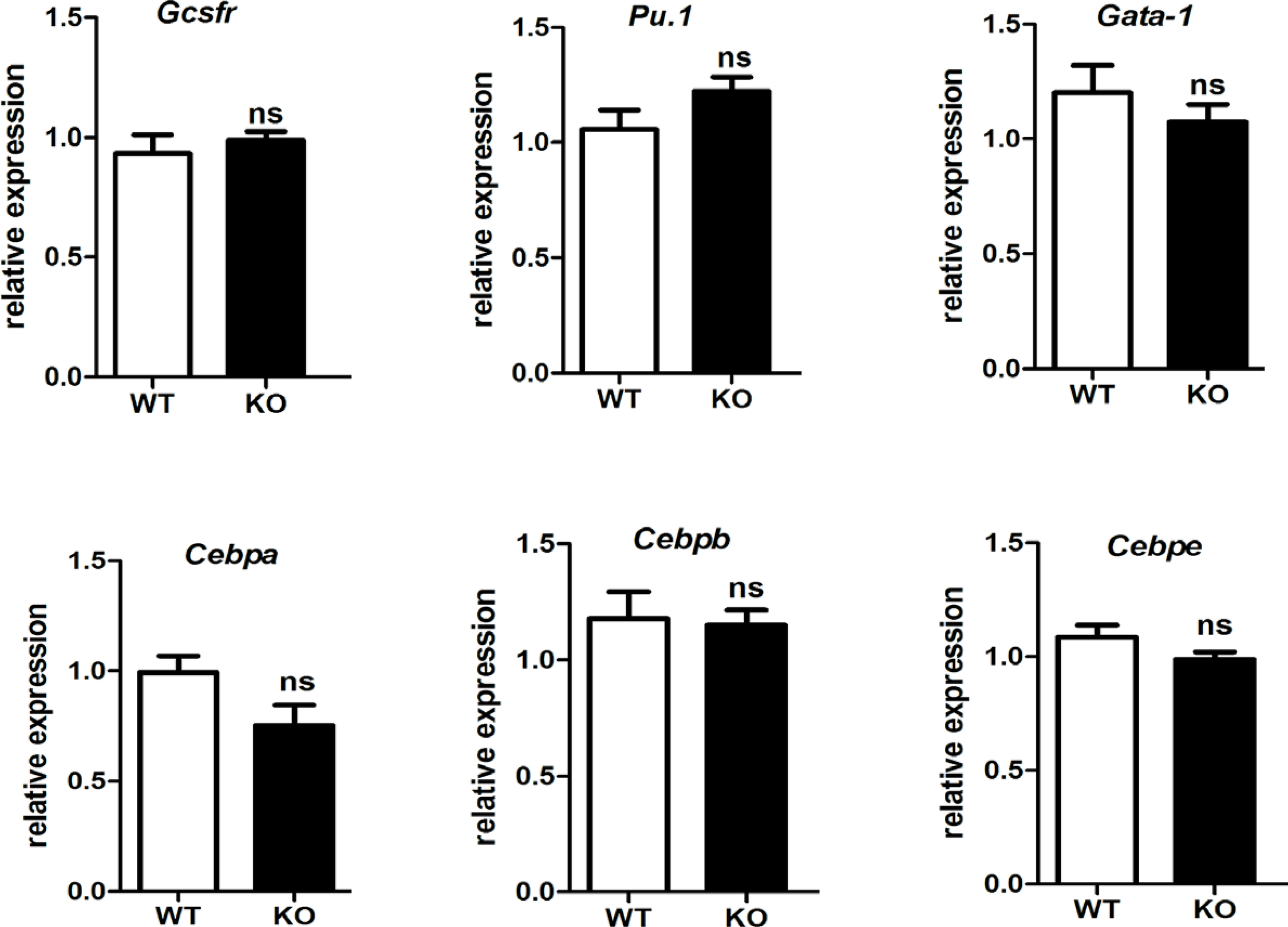

Supplement: S4 Fig — Bone marrow neutrophils were extracted RNA and determined the expression of Gcsfr, Pu.1, Gata-1, Cebpa, Cebpb and Cebpe by Real-tme PCR. Ns, none specific significance (Student’s t-test). (TIF) [file pgen.1007027.s004.tif]

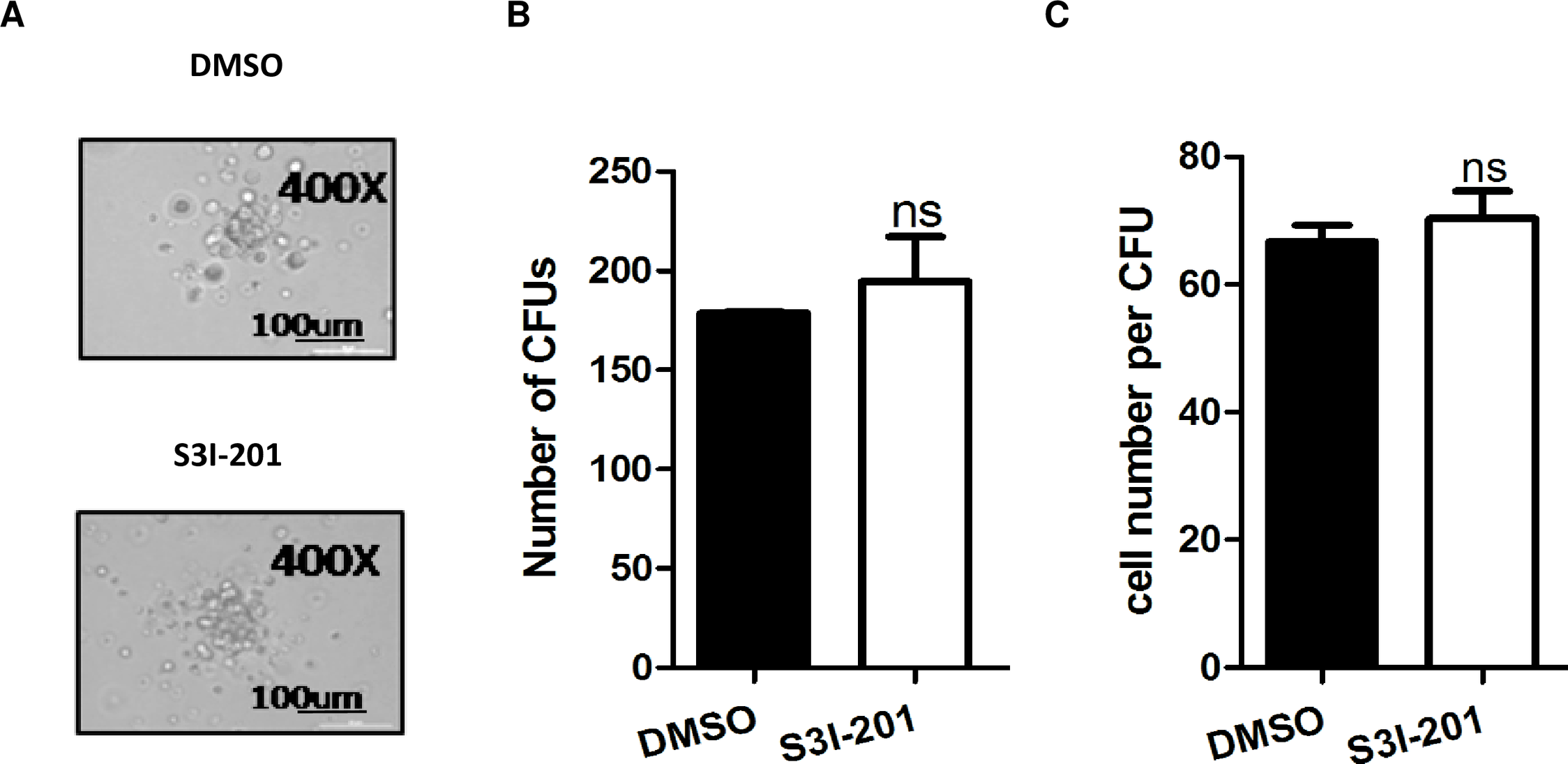

Supplement: S5 Fig — 1000 GMPs were sorted from MiR125a-/- bone marrow cells and then cultivated in G-CSF and S3I-201 or DMSO containing methylcellulose media. Photographed CFUs (A), colony numbers (B) and cell number per CFUs (C) were shown. Representative data were from three independent experiments. Ns, none specific significance (Student’s t-test). (TIF) [file pgen.1007027.s005.tif]

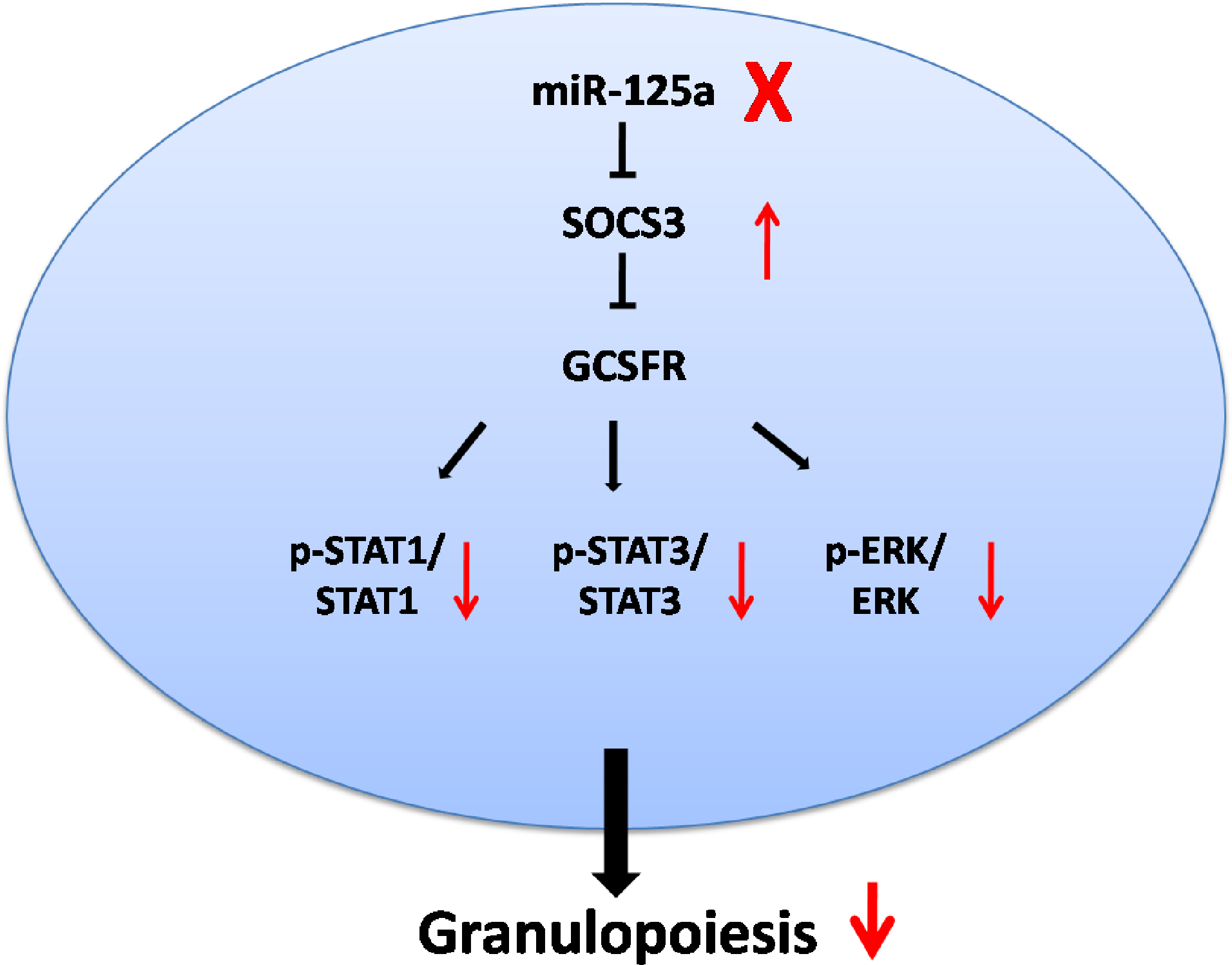

Supplement: S6 Fig — In wild-type granulocytes, miR-125a down-regulates the expression of SOCS3 that was leading to activation of STAT1, STAT3 and ERK. While in MiR125a -deficient granulocytes, the expression of SOCS3 was enhanced, weakening of STAT1, STAT3 and ERK activation and eventually reduced granulopoiesis. (TIF) [file pgen.1007027.s006.tif]

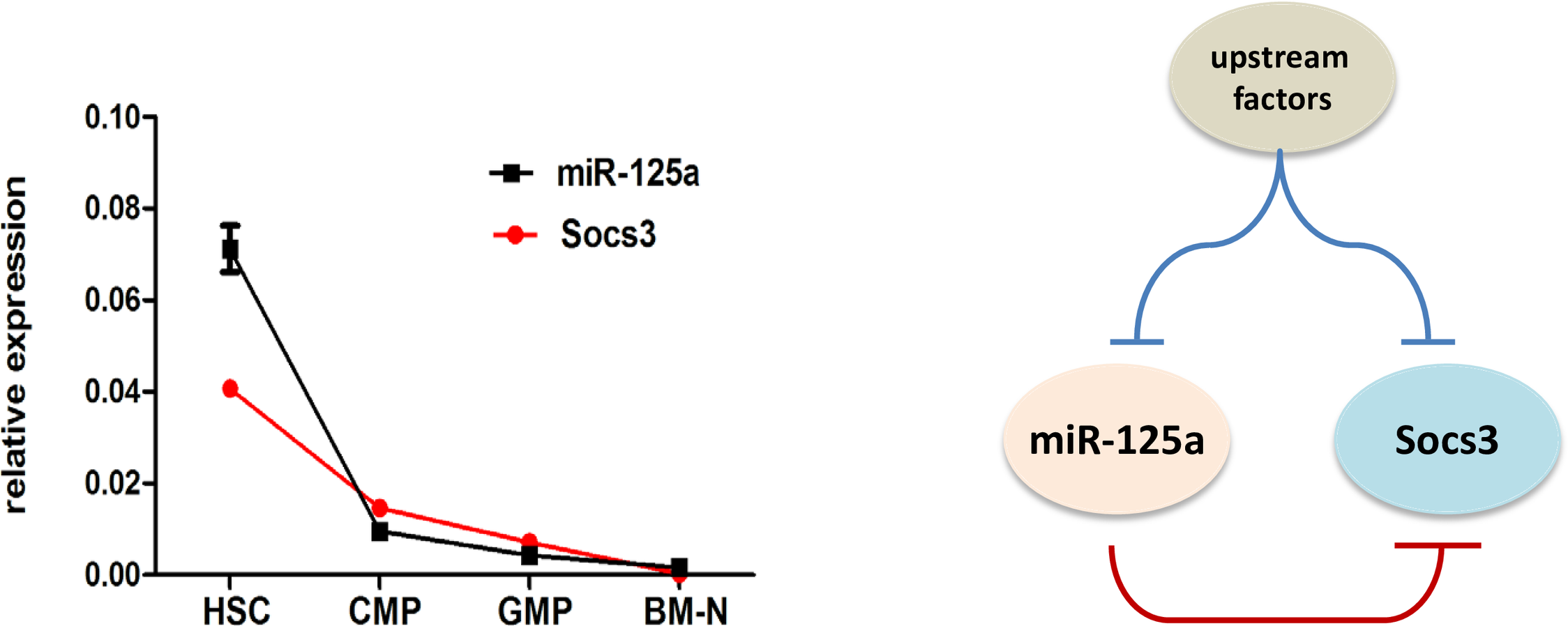

Supplement: S7 Fig — The expression of miR-125a and Socs3 mRNA was detected by real-time quantitative PCR (mean±s.d.,n = 3) (left). The regulation circuit of miR-125a and Socs3 during granulocyte development (right). (TIF) [file pgen.1007027.s007.tif]
